# More terminological clarity in the interprofessional field – a call for reflection on the use of terminologies, in both practice and research, on a national and international level 

**DOI:** 10.3205/zma001035

**Published:** 2016-04-29

**Authors:** Anika Mitzkat, Sarah Berger, Scott Reeves, Cornelia Mahler

**Affiliations:** 1 University Hospital Heidelberg, Department of General Practice and Health Services Research, Heidelberg, Germany; 2 Kingston University & St George's, University of London, Centre for Health & Social Care Research, London, United Kingdom

**Keywords:** Terminology, interprofessional relations, interprofessional health care teams, cooperative behaviour

## Abstract

The terminology which has been used up until now within interprofessional healthcare has been characterised by a certain definitional weakness, which, among other factors, has been caused by an uncritical adoption of language conventions and a lack of theoretical reflection. However, as terminological clarity plays a significant role in the development and profiling of a discipline, the clarification and definition of commonly-used terminology has manifested itself as a considerable objective for the interprofessional research community. One of the most important journals for research in the area of interprofessional education and care, the Journal of Interprofessional Care, has expanded its author guidelines relating to terminology, modeled after the conceptual considerations of the research group around Barr et. al and Reeves et al. A German translation of the suggested terms therein has been presented in this contribution, and discussed in light of the challenges to a possible adaptation for the German-speaking world. The objective is to assist communication in practice and research in becoming clearer, while promoting an increasing awareness to and the transparency of determined definitions and terminologies.

## Zusammenfassung

Die bisher verwendeten Begrifflichkeiten der interprofessionellen Gesundheitsversorgung zeichnen sich durch eine gewisse definitorische Schwäche aus, die unter anderem durch eine unkritische Übernahme von Sprachkonventionen und mangelnder theoretischer Reflexion bedingt ist. Da begrifflicher Klarheit jedoch eine wesentliche Funktion in der Entwicklung und Profilierung eines Faches zukommt, stellt sich die Klärung und Definition der zu verwendenden Terminologie als Aufgabe der interprofessionellen Forschungsgemeinschaft dar. Eines der wichtigsten Journals für wissenschaftliche Fragestellungen im Gebiet der interprofessionellen Bildung und Versorgung (Journal of Interprofessional Care) hat in Anlehnung an konzeptionelle Überlegungen der Forschergruppen H. Barr et al. und S. Reeves et al. die Autorenrichtlinie um eine Terminologie ergänzt. Eine deutsche Übersetzung der darin vorgeschlagenen Begriffe wird in diesem Beitrag vorgestellt und vor dem Hintergrund der Herausforderungen einer möglichen Adaption für den deutschsprachigen Raum diskutiert. Ziel ist es, die sprachliche Kommunikation auf dem Praxis- und Forschungsfeld zu mehr Deutlichkeit zu verhelfen und eine zunehmende Sensibilisierung und Transparenz für konsentierte Definitionen und Terminologien zu fördern.

## On the necessity of clarifying terminology

The necessity of a uniform, interprofessional terminology is axiomatic, as well as complex and challenging [1], [2]. One must know exactly what one is talking about. But who must know this and for what purposes? This is to be illustrated using an explanation of terms in medicine, and presented using examples of what this can mean in interprofessional contexts.

In medicine, as well as in other specialist professions, the difference between the profession-specific, academic, and the everyday, pragmatic use of language must be discussed in these regards. Within discipline-specific, academic language, the scientific community has agreed on rules and prerequisites which must exist for a term in order to be used in the correct way. Three different levels of linguistic communication in the healthcare professions are differentiated as part of this [3]: Firstly, national and international communication between specialists is of an academic nature. Here, termini technici, or technical terms in their entirety, with all applicable rules and premises, are assumed to be preexistent for all those involved in communication. (i.e. if “interprofessional teamwork” is being addressed, then it is clear to those involved in communication which underlying basic principles or concepts exist.) Secondly, the clinical, specialist use of terms, i.e. abbreviated and simplified oral or written communication, is often prevalent among specialists. Usually, a common understanding of the underlying concepts of a term may be assumed. At the same time, it must be noted that every abbreviation is accompanied by a loss of information and this may lead to misunderstandings, particularly when it has not been ascertained that basic principles have been established. (i.e. A tumor-board may inform others that it is working interprofessionally, which may trigger associations which indicate that different healthcare and social professions are working closely with one another, in close conjunction, mutually dependent and in a problem-orientated way. At the same time, this may simply mean that different professions, independently of each other, are examining a case and allowing their perspectives to be factored into the treatment process).

Communication between specialists and lay people has been identified as a third level. It goes without saying that because many specialist terms are also used in an everyday, pragmatic context, at this level, the definitions must accompany the terms to enable effective and comprehensible communication between all parties. (i.e. a nurse does not merely speak with their patient about an “ulcus ventriculi” for which a special diet must considered, but translates and explains that they have a stomach ulcer).

It must therefore be acknowledged that terminological clarity implies significant functions in the development and profiling of a profession. When it comes to everyday practice carried out by healthcare professionals, this means that phenomena may be named, recognised and taken into account according to their meaning in the course of the care-pathway. For the further development of a research disciple, this means that phenomena need to be conceptualised, and thereby made accessible for further research .

The area of interprofessional education and collaboration is continuously developing. A prerequisite for successful communication in research and practice is the widespread usage of a uniform terminology and language. Currently, within the German-speaking world, there are a multitude of terms which are not used in a uniform, consistent manner [4]. 

If one notes the different levels and purposes of specialist language communication, it becomes obvious that terminological clarifications do not necessarily come about themselves, but are more so subject to methodical terminology work through intensive discourse among specialists in the field. 

In the English-speaking world, the *Journal of Interprofessional Care* (JIPC) started the discussion on and demand for a clarification of terminology [5], [6], [7] insofar that it expanded its author guidelines by a section on the terminology to be used in manuscripts which are to be published in the Journal of Interprofessional Care [http://www.tandfonline.com/action/authorSubmission?journalCode=ijic20&page=instructions#.Vsrd3CwwfGE cited 2016-29-2]

A German translation of the terminology suggested is being presented in this article. The objective is to encourage this discussion in the German-speaking world and therefore to achieve more clarity in the communication in this area of practice and research.

## Side note: The challenge of adapting international terms for the German-speaking world

As a preliminary remark to the subsequent translation, it must be noted that different language conventions have evolved for the naming of different professional groups in the German healthcare system and in the English-speaking world. This manifests a challenge in the translation and adaptation of terms into German. While the English term “health and social professions” includes the profession of doctors within healthcare professions, in the German-speaking world, it is common to speak of “Medizin und Gesundheits(-fach-)berufen” [medical and healthcare (specialist) professions]. This German differentiation is disadvantageous in two respects. Firstly, it intertwines different categories insofar as it equates one discipline (medicine) with different practices (healthcare professions). Taxonomically, the correct differentiation would be: “Medizin und Gesundheitswissenschaften” [medicine and health sciences] (on a discipline level) or “Arzt-und Gesundheitsberufe” [medical and healthcare professions] (for the practice level). Secondly, it may be asked, with respect to this correction of taxonomy, in what sense the medical profession is NOT a healthcare profession and therefore why it should not be included under this term. 

Furthermore, different international educational and healthcare structures can lead to problems in translation. In many countries, most healthcare professions are taught at universities. This may then potentially lead to another understanding of terms within healthcare structures. 

In the following, the professions within the areas of medicine, nursing, therapy, diagnostics, etc. are included in the term “healthcare professions,” as demonstrated in the position paper of the GMA committee “Interprofessional Education in the Healthcare Professions” [8].

## German translation of the terminology suggested by the Journal of Interprofessional Care

Hereafter, the translation of terms and definitions from the chapter “terminology” from the author guidelines of the *Journal of Interprofessional Care (JIPC)* are presented. The terms were translated by JM and discussed and revised in an iterative process by CM, SB and AM. All participants are closely involved in the bachelor programme “Interprofessional Healthcare B.Sc.” as well as in interprofessional modules with medical students at the Medical Faculty of the University of Heidelberg (terminology see attachment 1 ). 

## Conclusions

In order to establish interprofessional structures and to carry out research in an increasingly complex healthcare system, a common language is indispensable. 

The terminology suggested by the *Journal of Interprofessional Care* brings light to the multitude of potential constellations for different professional groups within the healthcare system. Without doubt, an advantage lies, in the clarification of the commonly synonymous used terms discipline and profession on the one hand, and a description of potential relationships between them on the other. Conceptually, however, the terminology extends beyond pure description insofar as the differentiation of “cooperation,” “coordination,” “network” and “teamwork” refers to the respectively required structures within health care: For effective and efficient teamwork, identification with the group and their objectives must be provided. This has direct normative effects on interprofessional teaching and research, as based on the current state of research, this identification does not establish itself by nature, it requires specific competencies, as well as the appropriate structures These will have an effect not only on patientcare, but also on health economic and political decisions. The differentiation in relation to health care structures was a challenge for the translation into German.

The problems of adapting international terminologies for use in the German language context (research and practice) have been presented. This challenge is on the one hand, the result of different language conventions, and on the other hand, the result of the heterogeneity of national educational and healthcare systems. These differences and specific characteristics of the German healthcare system should be taken into account when compiling a German terminology of interprofessional terms. At the same time, international comparability plays an important role within the context of academic debates, and evaluation of interprofessional education and collaboration. This must also be considered and taken into account. 

The authors propose an extensive debate on the translated and terms presented here. They hope for an increasing awareness and reflection of researchers, practitioners, teachers and students when using the suggested terms.

## Acknowledgements

We wish to thank Johanna Mink for assisting us with the first translation of the terminology.

## Competing interests

Scott Reeves is Chief Editor and Cornelia Mahler is Associate Editor of the Journal of Interprofessional Care.

## Supplementary Material

"Terminology" from the author guidelines of the JIPC
