# Supplementary material for: More terminological clarity in the interprofessional field – a call for reflection on the use of terminologies, in both practice and research, on a national and international level
Source: GMS J Med Educ. 2016 Apr 29;33(2):Doc36. doi: 10.3205/zma001035 (PMC4895843; doi:10.3205/zma001035)
Supplement: "Terminology" from the author guidelines of the JIPC [file JME-33-36-s-001.pdf]

Terminology

Given the ongoing terminological uncertainty within the interprofessional field, the Journal now employs a range of key terms – each with an associated definition.[1] Submitting authors therefore need to ensure they select term(s) that best describes the activities presented in their work. They should also ensure they use the selected term(s) consistently:

Disciplines are regarded as broad academic fields, with examples including, anthropology, economics, geography, social science and political science.

Interdisciplinary teamwork is an approach like interprofessional teamwork (see below) but differs as the team members are composed of individuals from different disciplines such as psychology, anthropology, economics and geography.

Interprofessional collaboration involves different health and social care professions who regularly come together to negotiate and agree how to solve complex care problems or provide services. It differs from interprofessional teamwork as colleagues do not share a team identity and work together in a less integrated and interdependent manner.

Interprofessional coordination involves different health and social care professions whose work together is focused on coordinating care tasks between one another. It is also seen as a more 'limited' working arrangement as unlike collaboration, interprofessional communication may be less frequent in nature.

Interprofessional education occurs when members (or students) of two or more health and/or social care professions engage in learning with, from and about each other to improve collaboration and the delivery of care.

Interprofessional learning is learning arising from interaction between members (or students) of two or more professions. This may be a product of interprofessional education or happen spontaneously in the workplace or in education settings and therefore be serendipitous in nature.

Interprofessional networks are loosely organised groups of individuals from different health and social care professions who meet and work together on a periodic basis.

Interprofessional teamwork involves different health and/or social professions who, for example, share a team identity and work closely together in an integrated and interdependent manner to solve complex care problems and deliver services.

---

[1] These terms are based on glossaries published in: Barr H, Koppel I, Reeves S, Hammick M, Freeth D. Effective interprofessional education: Argument, assumption and evidence. Oxford: Blackwell; 2005 and also in: Reeves S, Lewin S, Espin S, Zwarenstein M. Interprofessional teamwork for health and social care. London: Blackwell-Wiley; 2010.

Intraprofessional is a term which describes any activity which is undertaken by individuals within the same profession.

Multidisciplinary teamwork is an approach where team members work alongside one another: in other words, parallel rather than interactive work. These types of teams are composed of different academic disciplines (social sciences, geography, economics) rather than from different health and social care professions such as medicine, nursing and social work.

Multiprofessional teamwork is an approach where team members work alongside one another: in other words, parallel rather than interactive work. These types of teams are composed of different health and social care professions.

Professions are occupational groups who in general provide services to others. Traditionally it has been used to describe the more established professions such as nurses, physicians or social workers. However, due to the on-going expansion of different roles in health and social care, it can also be employed to describe newer occupational groups; thereby providing a more inclusionary definition of this term.

Transdisciplinary practice occurs when an individual from one disciplinary group takes on a role(s) or task(s) of another disciplinary group. While these disciplinary activities are outside their usual scope of practice it is assumed they have the necessary expertise to complete them.

Transprofessional practice occurs when an individual from one professional group undertakes the role(s) or task(s) of another profession. While these professional activities are outside their usual scope of practice it is assumed they have the necessary expertise to complete them.

Unidisciplinary is an activity undertaken by one discipline alone.

Uniprofessional is an activity undertaken by one profession alone.

A final note on terminology

'Allied health' has been used as a 'catch-all' term to describe a diverse range of professional groups (i.e. occupational therapists, dietitians, physiotherapists). This is a misleading term as it assumes these different groups are homogeneous in some way and that they are also 'allied' to something. As a results we discourage its use. Instead, authors should list the different professional groups involved in their manuscripts.
